# High-grade B-cell lymphoma with *MYC* and *BCL2* rearrangements arising in a composite lymphoma

**DOI:** 10.1186/s13000-018-0714-z

**Published:** 2018-05-24

**Authors:** Alison M. Moore, Olga Moshkin, Gordon J. Swain, Susan Crocker, David P. LeBrun

**Affiliations:** 1 Department of Pathology and Molecular Medicine, Queen’s University, Kingston, ON Canada; 2 Peterborough Regional Health Centre, Peterborough, ON Canada

**Keywords:** Composite lymphoma, Double-hit lymphoma, High-grade B-cell lymphoma, Diffuse large B-cell lymphoma, Follicular lymphoma, Chronic lymphocytic leukemia, Small lymphocytic lymphoma, Cytogenetics, MYC, BCL2

## Abstract

**Background:**

We report the first case of composite lymphoma consisting of chronic lymphocytic leukemia/small lymphocytic lymphoma (CLL/SLL), follicular lymphoma (FL) and high-grade B-cell lymphoma with *MYC* and *BCL2* rearrangements within the same needle biopsy in which a clonal relationship between the FL and high-grade B-cell lymphoma components was demonstrated by molecular cytogenetics.

**Case presentation:**

An 85-year-old man presented with masses in his neck and right groin. Cutting needle biopsy of the inguinal mass revealed the three lymphoma types which were morphologically, immunophenotypically and topographically distinct. Fluorescence in situ hybridization (FISH) identified an *IGH-BCL2* rearrangement in both the FL and high-grade B-cell components while a *MYC* rearrangement was detected in the high-grade B-cell component alone.

**Conclusions:**

Our findings suggest that the high-grade lymphoma with *MYC* and *BCL2* translocations evolved through transformation of the FL by a process that entailed acquisition of the *MYC* translocation. No clonal relationship between the FL and CLL/SLL components was evident since the *IGH-BCL2* rearrangement was present in in the former but not the latter. This unique case of co-localized FL, CLL/SLL, and high-grade B-cell lymphoma contributes to our understanding of the clonal relationships that may exist between the components of composite lymphomas.

## Background

“Composite lymphoma” refers to the co-occurrence of two or more distinct lymphoma types at a single anatomical site [1]. Composite lymphomas are relatively rare, accounting for only 1–4% of lymphomas. Instances of histological transformation from an indolent non-Hodgkin lymphoma to more aggressive disease, typically diffuse large B-cell lymphoma (DLBCL), are often excluded from the composite lymphoma category even when the two components are present in the same sample. We describe a case of composite lymphoma consisting of chronic lymphocytic leukemia/small lymphocytic lymphoma (CLL/SLL), follicular lymphoma (FL) and high-grade lymphoma with *MYC* and *BCL2* rearrangements (formerly and more colloquially denoted “double-hit” lymphoma). All of these components were present in a single, small tissue sample obtained by cutting needle biopsy. To the best of our knowledge, the results derived from this case are unique and address important questions pertaining to the clonal interrelationships that exist between the three lymphoid neoplasms.

## Case presentation

An 85-year-old man presented with masses in his neck and right groin. CT confirmed bulky lymphadenopathy in the neck (4.1 × 3.5 cm) and right inguinal area (5.6 × 3.7 cm) and demonstrated extensive smaller lymphadenopathy as well as five liver masses measuring from 3.5 to 4.6 cm. The patient was otherwise asymptomatic with no B-symptoms. Biopsy of the inguinal mass with an 18 gauge cutting needle produced cores of tissue the largest of which measured 17 × 2 mm and contained elements of CLL/SLL, FL and DLBCL. Shortly after the biopsy, the patient presented to the Emergency Department with an acute upper gastrointestinal bleed and Hb of 59 g/L. Endoscopic gastric biopsy showed DLBCL. After 6 cycles of rituximab, cyclophosphamide, doxorubicin, vincristine and prednisone (R-CHOP) chemotherapy he had a dramatic response albeit with some residual lymphadenopathy. He therefore received another round of R-CHOP. Fifteen days later, he presented to the Emergency Department with dyspnea, fever and radiographic evidence of bilateral lung consolidation. His condition deteriorated rapidly despite aggressive antimicrobial and supportive therapy and he expired approximately 5 months after the original diagnosis.

**Fig. 1:**
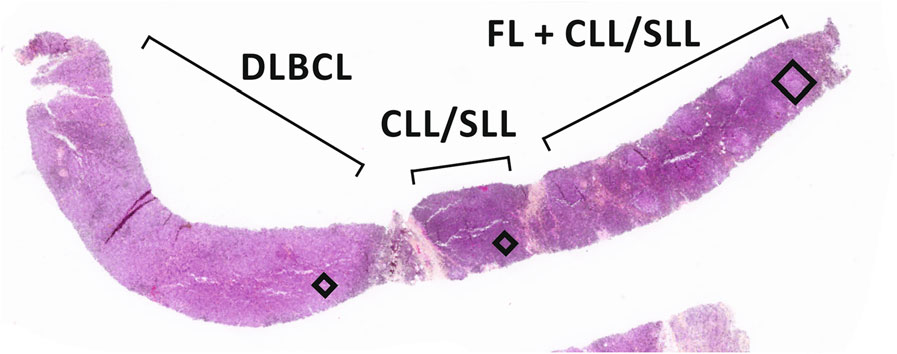
Core of biopsied tissue containing three distinct lymphoma types. DLBCL, diffuse large B-cell lymphoma; CLL/SLL, chronic lymphocytic leukemia / small lymphocytic lymphoma; FL, follicular lymphoma. Rectangles indicate where the images shown in Fig. 2 were captured

**Fig. 2:**
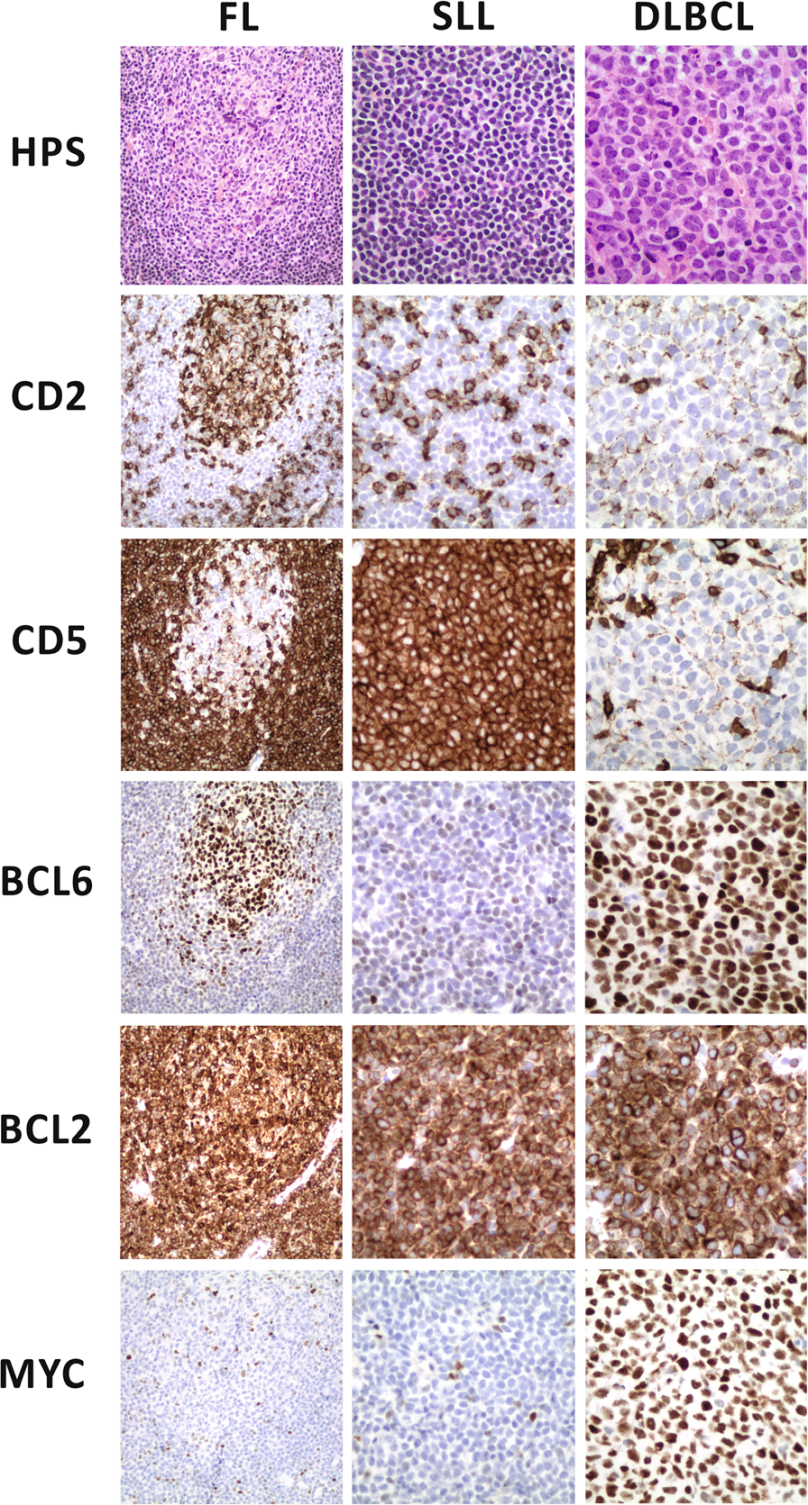
Morphological and immunohistological findings from three lymphoma types present in the same small biopsy sample. FL, follicular lymphoma; SLL, small lymphocytic lymphoma; and, DLBCL, diffuse large B-cell lymphoma. FL, original magnification 20×; SLL and DLBCL, original magnification 40×

The biopsy sample from the inguinal mass consisted of neoplastic lymphoid infiltrates with features of three distinct lymphoma types present in approximately equal proportions (Figs. 1 and 2). The FL component was at one end of the fragment and consisted of closely spaced lymphoid follicle centers containing a heterogeneous mixture of cells amongst which large centroblasts were sufficiently numerous to justify designation as grade 3A. These follicles were separated by diffuse sheets of small, round lymphocytes with an appearance characteristic of CLL/SLL. An adjacent area in this tissue fragment consisted of pure CLL/SLL without the FL component. At the opposite end of the core, adjacent to the deposit of pure CLL/SLL and separated from it by a thin band of fibrosis, were sheets of large centroblasts associated with numerous mitotic figures, karyorrhectic debris and scattered tingible body macrophages presenting an appearance typical of centroblastic DLBCL. Immunohistochemistry (IHC) performed on serial sections demonstrated the expected immunophenotype for each lymphoma type (Fig. 2 and Table 1). Specifically, all three lymphomas expressed CD20, CD79a and BCL2. The FL and DLBLC cells expressed BCL6 and CD10 but not CD5 or CD23. The CLL/SLL component expressed CD5 and CD23 but not BCL6 or CD10. And, the DLBCL, but not the other components, expressed Ki-67 and MYC in almost all neoplastic cells. These morphological and immunophenotypic findings unequivocally demonstrate the presence of all three lymphoma types in a single tissue fragment.

**Table 1:** Results from immunohistochemistry and FISH

Marker	FL^a^	CLL/SLL	DLBCL
CD20	+	+	+
CD79a	+	+	+
CD2	–	–	–
CD3	–	–	–
CD5	–	+	–
Cyclin D1	–	–	–
BCL6	+	–	+
CD10	+	–	+
CD23	–	+	–
BCL2	100%	100%	100%
MYC	10%	5%	~ 98%
Ki-67	60%	10%	~ 98%
FISH *IGH-BCL2*	RR	N	RR
FISH *MYC*	N	N	RR

^a^*FL* follicular lymphoma, *CLL/SLL* chronic lymphocytic leukemia/small lymphocytic lymphoma, *DLBCL* diffuse large B-cell lymphoma, *RR* rearranged, *N* normal

**Fig. 3:**
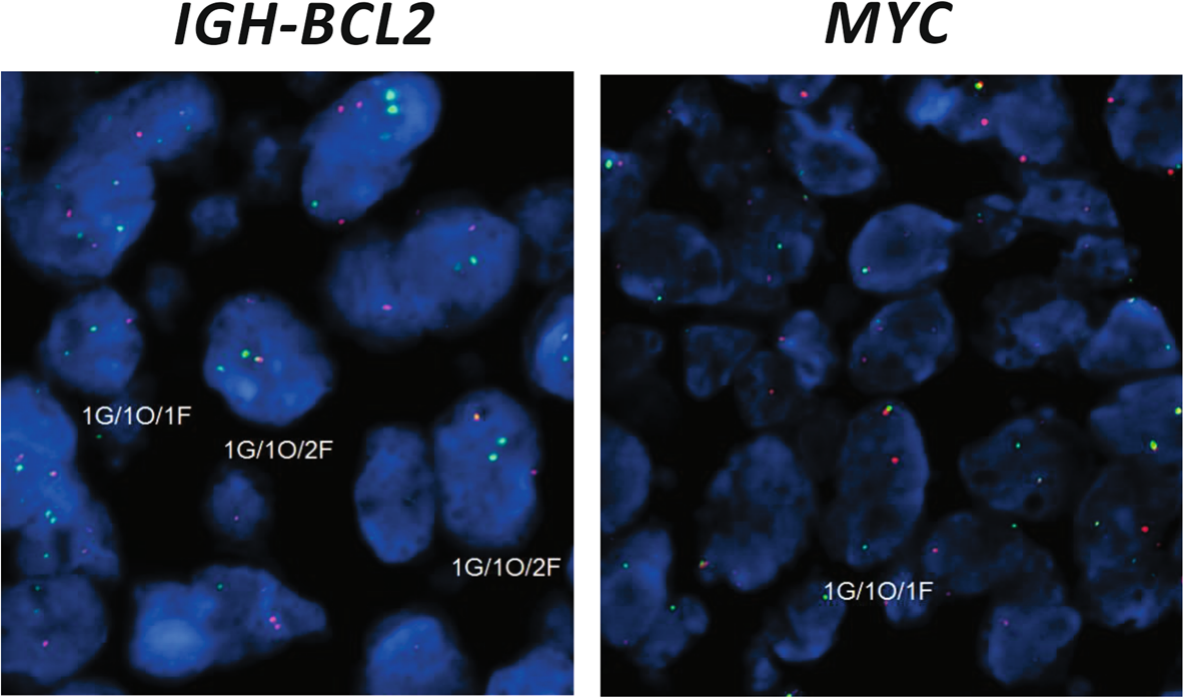
FISH results from the diffuse large B-cell lymphoma component. *IGH-BCL2* fusion (left) is demonstrated by the presence of fusion signals whereas *MYC* disruption (right) is shown by separate green and orange signals. G, green; O, orange; F, fusion

Fluorescence in situ hybridization (FISH) demonstrated the presence of an *IGH-BCL2* fusion in both the DLBCL and FL components, but not the CLL/SLL component, whereas *MYC* was disrupted in the DLBCL, but not the FL, component (Fig. 3). Considering these cytogenetics findings in the context of the revised 2017 WHO classification system, the DLBCL component should be designated “high-grade B-cell lymphoma with *MYC* and *BCL2* rearrangements”.

## Discussion

We are aware of published reports of ten previous cases of composite lymphoma that contained elements of FL and SLL/CLL [2–6]. In two of these, the biopsy sample also contained DLBCL [5, 6]. FL and CLL/SLL are generally considered indolent and are susceptible to transforming to more aggressive disease, most often DLBCL; in the case of CLL/SLL this occurrence is denoted “Richter’s Syndrome” [7, 8]. In cases of transformation, the DLBCL component either evolves directly from the pre-existing FL or CLL/SLL clone or from a common precursor clone from which both the indolent lymphoma and DLBCL subsequently diverge [7]. Therefore, initially it seemed likely that the DLBCL component in our case arose through transformation from either the FL or CLL/SLL components. Among the three reported cases (including ours) of composite lymphoma with FL, SLL and DLBCL elements, ours is the only one in which an *IGH-BCL2* fusion demonstrable by FISH was available to support the inference that the DLBCL component evolved directly from FL. This finding agrees with those of diSibio and colleagues who demonstrated the presence of an *IGH-BCL2* fusion in both the FL component of a composite lymphoma and a DLBCL that arose 15 years later [4]. Our case is also the first in which FISH demonstrated the presence of a *MYC* rearrangement in the DLBCL, but not the FL, component. *MYC* rearrangements are present in 25 to 50% of transformed follicular lymphomas and most of these rearrangement events occur during transformation. [9, 10] Especially given that *MYC* rearrangements are identifiable in only 5 to 10% of de novo DLBCLs, we feel justified in suggesting that the acquisition of the *MYC* rearrangement may have contributed functionally to transformation of FL to DLBCL. [11] DLBCLs with both an *IGH-BCL2* and a *MYC* rearrangement, currently denoted “high-grade B-cell lymphoma with *MYC* and *BCL2* rearrangements”, are associated with poor clinical outcomes [12]. Ours is the first reported case of composite lymphoma that includes elements of FL, CLL/SLL and high-grade B-cell lymphoma with *MYC* and *BCL2* rearrangements.

Published studies have addressed the clonal relationship between the FL and SLL/CLL components in cases of composite lymphoma. Use of the polymerase chain reaction (PCR) with primers for the *IGH* locus has demonstrated that the FL and CLL/SLL components contained different clonal rearrangements in 3 composite lymphomas, suggesting that these two components either arose from different clones *ab initio* or diverged prior to *IGH* gene rearrangement [2, 3]. Also in support of a separate clonal origin, FISH was used to demonstrate *IGH-BCL2* fusion in the FL, but not the SLL/CLL, component in an additional 2 cases (including ours) [4]. In contrast, Zhang and colleagues reported results from *IGH* PCR that suggested clonal identity between the FL and SLL components in 3 cases of composite lymphomas [5]. Therefore, although uncertainty persists, the bulk of available evidence currently favors a separate clonal origin for the FL and CLL/SLL components in composite lymphomas.

The relative rarity and pathological complexity of composite lymphomas makes treatment decisions challenging. In general, treatment is directed against the more aggressive component; in our case, this is high-grade B-cell lymphoma [1]. Standard first-line treatment for DLBCL is R-CHOP [12]. “Double-hit” status is associated with an elevated risk of treatment failure. While there has been interest in the potential utility of more intensive regimens to induce remission, such as dose-adjusted etoposide, prednisone, vincristine, cyclophosphamide, doxorubicin and rituximab (EPOCH-R), it is not clear that these improve overall survival [12]. Moreover, the toxicity of more intensive therapies limits their application for elderly patients or those with co-morbidities, both of which are prevalent amongst patients with composite lymphoma.

## Conclusions

The findings from our unique case of composite lymphoma in which FL, SLL and high-grade B-cell lymphoma with rearrangement of *MYC* and *BCL2* co-existed within a single, small tissue fragment suggest that the FL and SLL/CLL processes represent distinct neoplastic clones and that the high-grade B-cell component emerged through direct clonal evolution from the FL in a process that entailed acquisition of an *IGH-MYC* rearrangement.

## Abbreviations

CLL/SLL: Chronic lymphocytic leukemia / small lymphocytic lymphoma
CT: Computed tomography
DLBCL: Diffuse large B-cell lymphoma
EPOCH-R: Etoposide, prednisone, vincristine, cyclophosphamide, doxorubicin and rituximab
FISH: Fluorescence in situ hybridization
FL: Follicular lymphoma
IHC: Immunohistochemistry
PCR: Polymerase chain reaction
R-CHOP: Rituximab, cyclophosphamide, doxorubicin, vincristine and prednisone
